# Effects of Strength Training on Body Composition, Physical Performance, and Protein or Calcium Intake in Older People with Osteosarcopenia: A Meta-Analysis

**DOI:** 10.3390/nu17172852

**Published:** 2025-09-02

**Authors:** Jordan Hernandez-Martinez, Braulio Henrique Magnani Branco, Edgar Vasquez-Carrasco, Izham Cid-Calfucura, Tomás Herrera-Valenzuela, Eduardo Guzmán-Muñoz, Pedro Delgado-Floody, Yeny Concha-Cisternas, Pablo Valdés-Badilla

**Affiliations:** 1 Department of Physical Activity Sciences, Universidad de Los Lagos, Osorno 5290000, Chile; jordan.hernandez@ulagos.cl; 2 Department of Education, Faculty of Humanities, Universidad de la Serena, La Serena 1700000, Chile; 3 Graduate Program in Health Promotion, Cesumar University (UniCesumar), Maringá 87000-000, Brazil; braulio.branco@unicesumar.edu.br; 4 School of Occupational Therapy, Faculty of Psychology, Universidad de Talca, Talca 3465548, Chile; edgar.vasquez@utalca.cl; 5 Centro de Investigación en Ciencias Cognitivas, Faculty of Psychology, Universidad de Talca, Talca 3465548, Chile; 6 VITALIS Longevity Center, Universidad de Talca, Talca 3465548, Chile; 7 Department of Physical Activity, Sports and Health Sciences, Faculty of Medical Sciences, Universidad de Santiago de Chile (USACH), Santiago 8370003, Chile; izham.cid@gmail.com (I.C.-C.); tomas.herrera@usach.cl (T.H.-V.); 8 School of Kinesiology, Faculty of Health, Universidad Santo Tomás, Talca 3460000, Chile; eguzmanm@santotomas.cl (E.G.-M.); yenyf.concha@gmail.com (Y.C.-C.); 9 School of Kinesiology, Faculty of Health Sciences, Universidad Autónoma de Chile, Talca 3460000, Chile; 10 Department of Physical Education, Sport, and Recreation, Universidad de La Frontera, Temuco 4811230, Chile; pedro.delgado@ufrontera.cl; 11 Vicerrectoría de Investigación e Innovación, Universidad Arturo Prat, Iquique 1100000, Chile; 12 Department of Physical Activity Sciences, Faculty of Education Sciences, Universidad Católica del Maule, Talca 3460000, Chile; 13 Sports Coach Career, Faculty of Life Sciences, Universidad Viña del Mar, Viña del Mar 2520000, Chile

**Keywords:** muscle strength, aging, walking speed, geriatric assessment, nutrients

## Abstract

**Objective:** this systematic review with a meta-analysis aimed to evaluate the available body of published peer-reviewed randomized controlled trial (RCT) studies on the effects of different doses and types of strength training (ST) on body composition, physical performance, and protein or calcium intake in older people with osteosarcopenia. **Method:** a systematic literature search was conducted between July 2024 and August 2025 using five databases: PubMed, Medline, CINAHL Complete, Scopus, and Web of Science. PRISMA, TESTEX, RoB 2, and GRADE tools assessed methodological quality and certainty of evidence. Hedge’s g effect sizes were calculated for the abovementioned variables for the meta-analysis. **Results:** the protocol was registered in PROSPERO (code: CRD42025643858). Of 141 registers, seven RCTs with 349 participants were included. Seven overall and two subgroup meta-analyses showed significant increases in skeletal muscle mass index (SMMI; *p* < 0.01), maximal isometric handgrip strength (MIHS; *p* = 0.03), and protein intake (*p* = 0.03). There were no significant differences in bone mineral density (BMD), body fat percentage (BFP), gait speed, and calcium intake. However, meta-analysis by subgroups showed significant decreases in BFP *(p* = 0.01) in favor of elastic band training versus resistance training, with no significant differences in BMD. **Conclusions:** ST in older people with osteosarcopenia conditions increases SMMI, MIHS, and protein intake.

## 1. Introduction

Aging entails substantial physiological changes that favor the emergence of geriatric syndromes, such as sarcopenia, defined as loss of skeletal muscle mass index (SMMI) together with alterations in functional physical capacity [1], and osteopenia is defined as a decrease in bone mineral density (BMD) below normal reference values [2]. The standard method for detecting these alterations in body composition is dual energy X-ray absorptiometry (Dexa) and, secondarily, electrical bioimpedance [3]. In people over 60 years of age, an approximate annual decline of 1–1.5% in BMD, 1% in SMMI, and between 2.5 and 3% in muscle strength has been reported, a combination of decreased muscle mass and BMD [4]. Although these conditions have adverse consequences independently, their coexistence, known as osteosarcopenia, constitutes a more severe clinical phenotype. This is associated with an increased fall risk, fractures, loss of functionality, dependency, and high healthcare costs, exceeding the impact of each condition separately [5]. Because of this geriatric syndrome’s high healthcare expenditures, it is crucial to implement interventions to address these markers [5]. Given the high health burden, it is essential to develop and implement integrated interventions that simultaneously address bone and muscle health in older people [5].

Different therapies have been used to intervene in older people with osteosarcopenia, both pharmacological and non-pharmacological, with physical activity being a fundamental therapy [6]. So far, there are no specific pharmacological interventions to treat osteosarcopenia; however, medicines such as denosumab have shown promising effects on muscle mass and BMD [6]. In a study conducted by Bonnet et al. [7], in females with sarcopenia for 3 years, denosumab was compared with zoledronic acid or alendronate, presenting significant improvements in maximal isometric handgrip strength (MISH; *p* < 0.05), BMD (*p* < 0.01) and appendicular lean mass (*p* < 0.05), which is in favor of denosumab in comparison to zoledronic acid and alendronate. Another alternative to treat the decrease in SMMI, BMD, and muscle quality is the intake of supplements such as macronutrients (proteins and calcium) [8,9,10]. A descriptive associative study conducted by Yao, et al. [11] demonstrated an association of higher calcium intake with higher BMD in the lumbar region in Chinese older people. A literature review conducted by Putra, et al. [12] in older people shows that protein intake through supplementation of both animal and vegetable origin contributes to an increase in SMMI and an improvement in muscle quality, which helps prevent and combat sarcopenia in this age group. Another alternative for treating osteosarcopenia is physical activity, whether combined with macronutrient supplementation [13,14] or physical activity alone [15].

On the other hand, prescribing physical activity as a non-pharmacological intervention has proven to be an effective natural method in improving alterations in muscle mass, BMD, and physical performance in older people with osteosarcopenia [15]. In a meta-analysis conducted by Liu and Lee [16] in older females with osteosarcopenic obesity with 12-year interventions using elastic band training (EBT), there were significant increases in BMD (*p* < 0.001) and SMMI (*p* < 0.001), along with significant decreases in body fat percentage (BFP; *p* < 0.001) and gait speed (*p* < 0.001), in favor of EBT compared to inactive control groups. Similarly, in a meta-analysis conducted by Yang, et al. [17] in older people with osteosarcopenic obesity through 12-week strength training (ST) interventions, there were significant increases in BMD (*p* = 0.03), along with significant decreases in BFP (*p* = 0.02) and in timed chair stand test (*p* = 0.02), in favor of ST compared to inactive control groups. However, there were no significant increases in SMMI (*p* = 0.38) in favor of either group.

Although there is evidence of the positive effects of physical activity as a non-pharmacological treatment, mainly for ST on body composition and physical performance in older people with osteosarcopenia [7,15,16,17], the impact of ST at different training doses and in subgroup analyses of ST on body composition, physical performance, and macronutrient intake in older people with osteosarcopenia are so far unknown. Likewise, it is important to update the scientific evidence in systematic reviews with meta-analyses in the clinical setting [18,19]. Therefore, this meta-analysis aimed to analyze the available body of published peer-reviewed randomized controlled trials (RCTs) on the effect of different doses and types of ST on primary outcomes, such as body composition, and secondary outcomes, such as physical performance and protein or calcium intake, in older people with osteosarcopenia.

## 2. Methods

### 2.1. Protocol and Registration

The PRISMA guidelines were adhered to in this systematic review with a meta-analysis [20], and its protocol is registered in PROSPERO (the International Prospective Register of Systematic Reviews) under the ID CRD42025643858.

### 2.2. Eligibility Criteria

The inclusion criteria for this systematic review with a meta-analysis were met by original, peer-reviewed articles published until August 2025 that were unrestricted by language or publication date. The materials excluded were conference abstracts, books and book chapters, editorials, letters to the editor, protocol records, reviews, case studies, and trials. In addition, this systematic review used the PICOS (population, intervention, comparator, outcome, and study design) framework (see Table 1).

### 2.3. Information Search Process and Databases

The search process was conducted between July 2024 and August 2025 using five generic databases: PubMed, Medline, CINAHL Complete, Scopus, and Web of Science (core collection). The US National Library of Medicine Medical Subject Headings (MeSH) used free language terms related to ST, body composition, physical performance, and macro- and micronutrient intake in older people with osteosarcopenia. The search string used was as follows: (“osteosarcopenia” OR “osteosarcopenic” OR “osteo-sarcopenic” OR “sarco-osteopenic” OR “sarco-osteoporosis”) AND (“resistance training” OR “resistance exercise” OR “strength training” OR “elastic band training”) AND (“body composition” OR “body fat” OR “fat-free mass” OR “fat mass” OR “muscle mass” OR “body mass index” OR “nutritional status” OR “bone mineral density” OR “skeletal muscle mass index”) AND (“physical performance” OR “physical function” OR “physical fitness” OR “functional independence” OR “functional dependence” OR “functional mobility” OR “health condition” OR “falls” OR “fall risk” OR “risk of fall” OR “falling risk” OR “balance” OR “static balance” OR “dynamic balance” OR “walking speed” OR “gait speed” OR “mobility” OR “strength” OR “muscle strength” OR “upper body strength” OR “lower body strength” OR “muscle power”) AND (“elderly” OR “older people” OR “older adults” OR “older subject” OR “aging” OR “ageing” OR “aged”). To assist in locating additional relevant studies, two independent experts were consulted on the included publications and the inclusion and exclusion criteria. We stipulated two requirements for the experts: (i) to hold a PhD in sport science and (ii) to have peer-reviewed publications on physical activity in various population groups and/or physical performance published in journals with an impact factor according to Journal Citation Reports^®^. We did not disclose our search strategy to specialists to avoid bias in their searches. After completing these steps, we searched a database on 10 August 2025, for relevant retractions or errata related to the listed papers.

### 2.4. Studies Selection and Data Collection Process

Studies were exported using the EndNote reference manager (version X9, Clarivate Analytics, Philadelphia, PA, USA). Independent searches were conducted by JHM and ICC, who removed duplicates, screened titles and abstracts, and assessed full texts. No discrepancies were identified at this stage. The process was repeated for studies recommended by external experts and those identified through reference list searches. Full texts of potentially eligible articles were then reviewed, and the reasons for excluding studies that did not meet the selection criteria were documented.

### 2.5. Methodological Quality Assessment

TESTEX, a tool for exercise-based intervention studies [21], assessed the chosen studies’ methodological quality. One potential exclusion criterion was TESTEX results [21]. According to Smart, Waldron, Ismail, Giallauria, Vigorito, Cornelissen and Dieberg [21], there is a 15-point rating system (5 points for study quality and 10 points for reporting). Two authors (JHM and ICC) carried out this process separately, while a third author (THV) served as a referee for cases that were on the borderline and needed further validation from another author (PVB).

### 2.6. Data Synthesis

The following data were obtained and analyzed from the selected studies: (i) author and year of publication; (ii) country of origin; (iii) study design; (iv) sex and mean age of the sample; (v) number of participants in the intervention and control groups; (vi) body composition assessment tool; (vii) diagnostic criteria for osteosarcopenia; (viii) training volume (total duration, weekly frequency, and time per session); (ix) training intensity; (x) primary outcome (body composition); (xi) secondary outcome (physical performance, and protein or calcium intake); (xii) adverse events; and (xiii) adherence.

### 2.7. Risk of Bias in Individual Studies

Two independent researchers (JHM and ICC) evaluated the risk of bias version 2 (RoB 2) of the included studies, and a third researcher (PVB) analyzed the results. The Cochrane Handbook for Systematic Reviews of Interventions’ recommendations for RCTs was the foundation for this evaluation [22]. Based on the randomization procedure, departures from the planned interventions, missing outcome data, outcome assessment, and choice of the reported result, the risk of bias was categorized as “high,” “low,” or “some concerns.”

### 2.8. Summary Measures for Meta-Analysis

The study methodology includes meta-analysis; complete information is available in PROSPERO (registration code: CRD42024614097). Meta-analyses were only performed in the present case when ≥3 studies were available [23]. Effect sizes (ES; Hedge’s g) for body composition, physical performance, and protein or calcium intake in the ST and control groups (CGs) were calculated using the pre-training and post-training means and SD (standard deviation) for each dependent variable. Data were standardized using the change score SD. The ES values are presented with 95% confidence intervals (95%CIs). Calculated ES was interpreted using the following scale: trivial: <0.2; small: 0.2–0.6; moderate: >0.6–1.2; large: >1.2–2.0; very large: >2.0–4.0; extremely large: >4.0 [24]. The random-effects model was used to account for differences between studies that might affect the effect of ST. Comprehensive meta-analysis software (Version 2.0; Biostat, Englewood, NJ, USA) was used. Statistical significance was set at *p* ≤ 0.05 [25] and was used to perform these calculations. In each trial, the random-effects model (Der Simonian–Laird approach) was used to calculate and pool the standardized mean difference (SMD) and mean difference (MD) of BFP, SMMI, BMD, MIHS dominant and non-dominant hand, gait speed, protein intake, and calcium intake (ST vs. CG). The fundamental premise of the random-effects model is that genuine effects (interventions and duration, among others) vary throughout studies and that samples are selected from populations with varying ES. The data were pooled if there were at least three studies that showed the same results [26].

Heterogeneity between trial results was tested with Cochran’s Q test [27] and the I^2^ statistic. I^2^ values of <25%, 25–50%, and >50% represent small, medium, and large amounts of inconsistency [28]. Egger regression tests were performed to detect small study effects and possible publication bias [29].

### 2.9. Sensitivity Analysis

To assess the robustness and stability of the results, sensitivity analyses were conducted for all meta-analyses that included at least four studies. A leave-one-out approach was applied, whereby the pooled ES was recalculated after sequentially excluding each individual study, using the same Der Simonian–Laird random-effects model applied in the main analysis. For each iteration, changes in the overall ES, statistical significance (*p*-value), and heterogeneity parameters (τ^2^, Q, and I^2^) were evaluated. In addition, targeted sensitivity analyses were performed by excluding studies identified as potentially problematic, based on two predefined criteria: (i) High risk of bias: Studies classified as “high risk” in the overall assessment using the RoB 2.0 tool were excluded. This analysis was only performed when their removal left at least three studies in the meta-analysis. (ii) Outlier or influential studies: Identified through a combination of visual inspection of forest plots and quantitative influence diagnostics [30]. A study was considered an outlier if its ES substantially deviated from the pooled confidence interval or if it exerted disproportionate influence on the overall estimate, as indicated by extreme values in one or more influence metrics. Specifically, the following thresholds were applied: DFBETAS > |1|, indicating substantial impact on model coefficients; Cook’s distance > 4/n, where n is the number of studies (values > 0.5 were also considered in small samples); and hat values > 2 k/n, where k is the number of predictors (typically k = 1 in random-effects models). Studies exceeding one or more of these thresholds were flagged and excluded in additional sensitivity analyses to assess the robustness of the pooled effects.

### 2.10. Moderator Analysis

Using a random-effects model and independent computed single-factor analysis, potential sources of heterogeneity likely to influence training effects were selected a priori.

### 2.11. Subgroup Analysis

Since adaptive responses to ST programs may be affected by the type of training (e.g., resistance training or EBT) [31], these factors were considered possible moderating variables.

### 2.12. Certainty of Evidence

Studies were categorized as having high, moderate, low, or very low confidence based on their assessment of the GRADE scale [32]. Because studies with RCT designs were included, all analyses began with a high degree of certainty and were downgraded if there were concerns about bias, consistency, accuracy, precision, directness of results, or risk of publication bias [32]. Two authors evaluated the studies separately (JHM and ICC), and any disagreements were settled by agreement with a third author (EVC).

## 3. Results

### 3.1. Study Selection

**Figure 1:**
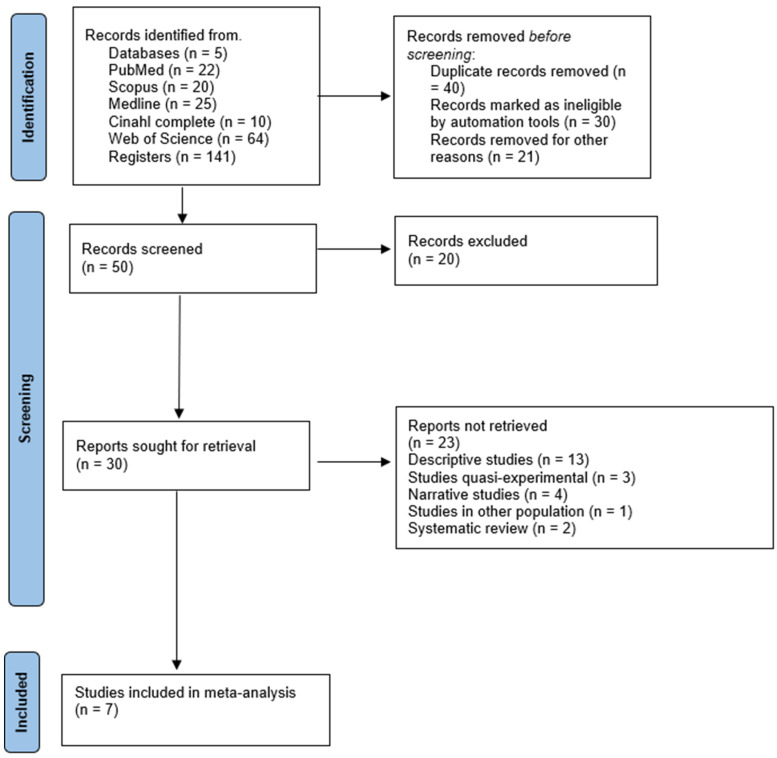
Flowchart of the review process. Legends: based on the PRISMA guidelines [20].

Figure 1 details the search process for the studies. A total of 141 records were found. Subsequently, duplicates were eliminated, and the studies were filtered by selecting the title, abstract, and keywords, resulting in 50 references. In the subsequent analysis phase, 20 studies were excluded because the texts did not meet the search criteria, leaving 30. Subsequently, thirteen studies were descriptive, two systematic reviews, three quasi-experimental studies, four narrative studies, and one study in other populations. Therefore, seven studies met all selection criteria [33,34,35,36,37,38,39].

### 3.2. Methodological Quality

The seven selected studies were analyzed using the TESTEX scale (Table 2). All the studies achieved a score equal to or higher than 60% on the scale [33,34,35,36,37,38,39], namely, 13/15 [35], 14/15 [34,36,37,38,39], and 15/15 [33]. 

### 3.3. Risk of Bias

**Figure 2:**
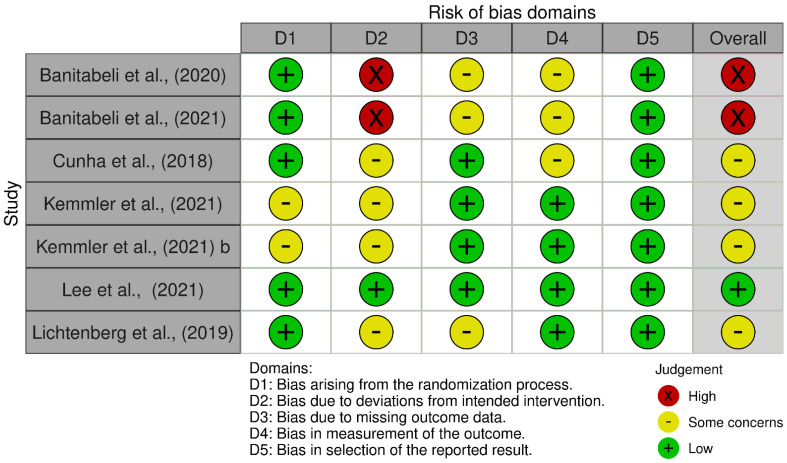
Risk of bias within studies. Legends: D1: randomization process; D2: deviations from the intended interventions; D3: missing outcome data; D4: measurement of the outcome; D5: selection of the reported result [33,34,35,36,37,38,39].

**Figure 3:**
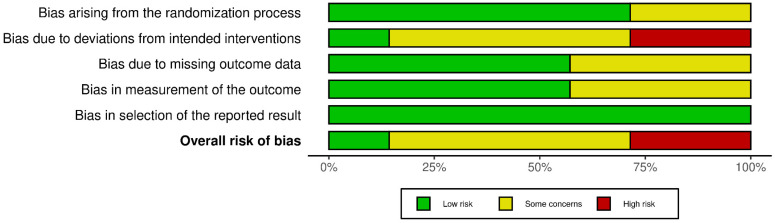
Risk of bias summary: review the authors’ judgments about each risk of bias item in each included study.

Among the included studies, only one was judged to have a low risk of bias [37], indicating that its methodological quality and internal validity were adequately safeguarded across all domains. In contrast, four studies were rated as raising some concerns [34,35,36,38], suggesting potential methodological limitations in specific domains, such as the randomization process, missing outcome data, or measurement of outcomes, which may have introduced a certain degree of uncertainty in the interpretation of their findings. Furthermore, two studies were identified as having a high risk of bias [32,33], mainly due to deviations from intended interventions and issues in the assessment outcomes, which could substantially affect the reliability of their results. Taken together, this distribution highlights that most of the evidence base is characterized by studies with at least some concerns regarding risk of bias, and the presence of high-risk studies further emphasizes the need for cautious interpretation of the overall findings. Figure 2 and Figure 3 provide a comprehensive summary of the research’s risk of bias.

### 3.4. Studies and Sample Characteristics

All studies were RCTs, of which three were conducted in Asia [33,34,38], three in Europe [36,37,39], and one in South America [35]. Four were conducted in females [33,34,35,38] and three in males [36,37,39], with a mean age of 71.3 ± 6.34 years and a total of 349 participants (188 in the experimental group and 161 in the CG). In the experimental groups, three of them performed ST interventions using elastic bands [33,34,38], while four studies performed ST interventions using free weights [35,36,37,39]. In all the ST interventions, upper and lower body exercises were performed. The duration of the interventions ranged from 12 to 18 months with frequencies of 1 to 3 sessions per week of 60 min duration with intensities between 50% and 85% of the one-repetition maximum [35,36,37,39] and 10 to 13 points on the OMNI Perceived Exertion Scale for Resistance Exercise (OMNI-RES) and Rate of Perceived Exertion (RPE) scales [33,34,38]. All the studies showed primary outcomes, and only one did not present secondary outcomes [35]. All studies had adherence than ≥85%, four studies did not observe adverse events following their interventions [36,37,38,39], and three did not report adverse events [33,34,35]. All these results are presented in detail in Table 3.

### 3.5. Meta-Analysis Results

The overall effects of ST on body composition, physical performance, and protein or calcium intake variables are shown in Table 4. Forest plots are shown in the Supplementary Materials (Figures S1–S7), (forest plot of changes in BMD, BFP, SMMI, MIHS, gait speed, protein intake, and calcium intake in older people with osteosarcopenia participating in ST compared with older people with osteosarcopenia assigned as controls). There were significant large effects (*p* < 0.05) in favor of ST in SMMI, MIHS, and protein intake (ES = 0.91 to 1.48). In BFP, BMD, gait speed, and calcium intake, there were no significant differences (*p* > 0.05) with moderate to large ES (ES = 0.20 to 0.64).

### 3.6. Meta-Analysis Results with Sensitivity Analysis

Regarding body composition, for BFP, the meta-analysis revealed a moderate but non-significant effect in favor of ST (ES = 0.644; *p* = 0.101; I^2^ = 89.1%). Sensitivity and influence analyses identified three studies, Kemmler, Schoene, Kohl and von Stengel [37], Banitalebi, Faramarzi, Ghahfarokhi, SavariNikoo, Soltani and Bahramzadeh [33], and Banitalebi, Ghahfarrokhi and Dehghan [34], as disproportionately impacting the overall ES and heterogeneity. In particular, Kemmler, Schoene, Kohl and von Stengel [37] showed high influence statistics (Cook’s distance = 0.7107, DFBETAS = –1.3045, hat value = 0.5201), exceeding commonly accepted thresholds. Banitalebi, Faramarzi, Ghahfarokhi, SavariNikoo, Soltani and Bahramzadeh [33] also displayed high values (Cook’s distance = 0.4760, DFBETAS = 0.8022, and hat value = 0.9240), while Banitalebi, Ghahfarrokhi and Dehghan [34] presented more moderate but still influential values (Cook’s distance = 0.0675, DFBETAS = 0.2276, and hat value = 1.6346). The exclusion of Banitalebi, Faramarzi, Ghahfarokhi, SavariNikoo, Soltani and Bahramzadeh [33] and Banitalebi, Ghahfarrokhi and Dehghan [34] was further justified by their classification as high risk of bias studies according to the RoB 2 tool. After excluding these three studies, the analysis yielded a statistically significant effect in favor of ST (ES = 0.511; 95% CI [0.073, 0.949]; *p* = 0.022), along with a substantial reduction in heterogeneity (I^2^ = 27.2%, τ^2^ = 0.041).

A similar pattern was observed for SMMI, where the exclusion of the studies by Kemmler, Schoene, Kohl and von Stengel [37] and Lee, Lee, Lin, Liao, Liou and Huang [38] also resulted in a statistically significant effect in favor of ST (ES = 1.446; 95% CI [0.642, 2.25]; *p* = 0.0004), with reduced heterogeneity (I^2^ dropped from 85.53% to 73.34% and τ^2^ from 0.6812 to 0.366). Both studies showed high influence metrics—Cook’s distance values of 0.5521 and 0.4770, DFBETAS of 0.9345 and –0.7508, and hat values of 0.1939 and 0.2014, respectively—supporting their disproportionate impact on the pooled estimate. For BMD, sensitivity and influence analyses did not identify any statistically influential studies. However, the studies by Banitalebi, Faramarzi, Ghahfarokhi, SavariNikoo, Soltani and Bahramzadeh [33] and Banitalebi, Ghahfarrokhi and Dehghan [34] were excluded due to their classification as high risk of bias according to the RoB 2 tool. After their exclusion, the meta-analysis showed a slight decrease in the ES (ES = 0.233; 95% CI [–0.093, 0.56]), which remained non-significant (*p* = 0.1616), and heterogeneity was fully eliminated (I^2^ = 0%, τ^2^ = 0).

Regarding macro- and micronutrient intake, the sensitivity and influence analyses for protein intake identified the study by Banitalebi, Faramarzi, Ghahfarokhi, SavariNikoo, Soltani and Bahramzadeh [33] as having a substantial impact on the pooled effect estimate. Its exclusion resulted in a marked reduction in heterogeneity (I^2^ decreased from 87.88% to 38.83%) while preserving a statistically significant overall effect (ES = 0.50; 95% CI [0.06, 0.94]; *p* = 0.0271). Influence diagnostics supported this observation, with the study showing a DFBETAS of 2.1310, a Cook’s distance of 0.8950, and a hat value of 0.2488, values indicating a disproportionate influence on the overall ES. For calcium intake, it is important to highlight that a sensitivity analysis could not be conducted due to the small number of studies included in the meta-analysis (n = 3). This methodological limitation reduced the ability to verify the consistency and reliability of the findings. As such, the results related to calcium intake should be interpreted with caution, and future studies with more robust designs and larger sample sizes are needed to reinforce these conclusions.

For the physical performance variables MIHS and gait speed, sensitivity analysis could not be performed due to the limited number of studies included in each comparison (n = 3). This methodological limitation restricted the ability to assess the robustness and stability of the pooled estimates. Therefore, the findings related to these outcomes should be interpreted with caution, and further research, including a greater number of high-quality studies, is needed to confirm and strengthen these preliminary results.

### 3.7. Meta-Analysis Subgroup

#### Subgroup Analysis by Type ST

In resistance training, loads were expressed in kilograms, either using free weights or variable load machines. In contrast, with elastic bands, the force was exerted through the tension of the bands used, which varied in color. All of the studios analyzed used the same model of elastic bands (Thera-Band^®^, The Hygienic Corporation, Akron, OH, USA), where these colors, like the tensions, are associated with kilograms [40,41]. In BMD, there were no significant differences in resistance training (*p* = 0.11) as in EBT (*p* = 0.16). However, in BFP, there were significant differences in favor of EBT (*p* = 0.01) without reporting significant differences in resistance training (*p* = 0.76). These results are presented in the Supplementary Materials (Figures S8 and S9), (forest plot of changes in subgroup BMD and BFP in older people with osteosarcopenia participating in ST compared with older people with osteosarcopenia assigned as controls). It is worth noting that, due to the limited number of studies included in each subgroup analysis (n = 3), sensitivity analyses could not be performed. This limitation restricts the ability to assess the robustness of the subgroup effects, and therefore, the findings should be interpreted with caution.

A comparative summary of the effects of EBT and resistance training across all outcomes is presented in Table 5, providing a concise overview of differences in direction, significance, and magnitude of changes between both training modalities.

### 3.8. Certainty of Evidence

The certainty of evidence was insufficient to support definitive recommendations on interventions ST in body composition, physical performance, and protein or calcium intake. Although some studies showed promising results, the findings highlight the need for further research to obtain more evident conclusions and guide evidence-based interventions in this population. Future trials should include larger sample sizes, longer follow-up periods, and standardized protocols to strengthen the quality of evidence. Moreover, addressing methodological limitations and ensuring rigorous study designs will be essential to clarify the true impact of ST interventions (Table 6).

## 4. Discussion

This systematic review with a meta-analysis aimed to analyze the available body of published peer-reviewed RCTs on the effect of different doses and types of ST on primary outcomes, such as body composition, and secondary outcomes, such as physical performance and protein or calcium intake, in older people with osteosarcopenia. Our meta-analysis identified large, significant effects (*p* < 0.05) in favor of ST in SMMI, MIHS, and protein intake (ES = 0.91 to 1.48). In BFP, BMD, gait speed, and calcium intake, there were no significant differences (*p* > 0.05) with moderate to large ES (ES = 0.20 to 0.64). According to ST, the subgroup analysis did not report significant differences in BMD for resistance training (*p* = 0.11) and EBT (*p* = 0.16). However, for BFP, there were significant differences in favor of EBT (*p* = 0.01), with no significant differences reported in resistance training (*p* = 0.76).

### 4.1. Body Composition

#### 4.1.1. Body Fat Percentage

Our meta-analysis did not find statistically significant improvements in BFP in favor of ST. However, four of the six experimental groups analyzed showed significant reductions in BFP, suggesting potentially meaningful changes from a clinical perspective. In older people with osteosarcopenia, even modest decreases in adiposity can lead to improvements in metabolic health, functional mobility, and a reduced risk of cardiometabolic comorbidities [42]. This is particularly relevant given the age-related decline in muscle mass and strength, as reducing excess fat mass may help optimize muscle quality and preserve functional independence. This is similar to the findings of Yang, Ye, Zhu, Zhang, Liu, Xie, Long, Huang, Niu, Luo and Wang [17] in a meta-analysis of older people with osteosarcopenic obesity who reported significant improvements in BFP in favor of ST (*p* = 0.02). Likewise, Liu and Lee [16], in a meta-analysis of older females with osteosarcopenic obesity, reported significant improvements in BFP in favor of ST (*p* < 0.001). From a mechanistic perspective, reductions in fat mass induced by ST are likely related to increases in total energy expenditure, improvements in insulin sensitivity, and the modulation of adipokines and inflammatory markers [42]. Enhanced muscle mass and strength contribute to higher resting metabolic rate, thereby favoring a healthier body composition profile [31]. Thus, beyond the statistical significance, these adaptations are meaningful in clinical terms, as they may delay functional decline and support independence in older people with osteosarcopenia. In this sense, based on Cunha, Ribeiro, Tomeleri, Schoenfeld, Silva, Souza, Nascimento, Sardinha and Cyrino [35] and our meta-analysis, a higher training volume (three sets per exercise) during ST may be necessary to induce significant improvements in BFP in older females with osteosarcopenia. Conversely, the sensitivity analysis revealed that the exclusion of the studies by Kemmler, Schoene, Kohl and von Stengel [37], Banitalebi, Faramarzi, Ghahfarokhi, SavariNikoo, Soltani and Bahramzadeh [33], and Banitalebi, Ghahfarrokhi and Dehghan [34] had a disproportionate impact on the overall effect estimate and heterogeneity. After removing these three studies, improvements were observed in both the precision and statistical significance of the effect. Consequently, the results of the sensitivity analysis strengthen the evidence that ST can lead to significant improvements on BFP in older people with osteosarcopenia, provided that studies with high risk of bias or extreme effects distorting the overall estimate are excluded.

#### 4.1.2. Skeletal Muscle Mass Index

Significant improvements in SMMI in favor of ST were found. This is in line with that reported in a recent meta-analysis by Liu and Lee [16] in older females with osteosarcopenic obesity who reported significant improvements in SMMI in favor of ST (*p* < 0.001). However, this differs from that reported in a meta-analysis by Yang, Ye, Zhu, Zhang, Liu, Xie, Long, Huang, Niu, Luo and Wang [17] in older people with osteosarcopenic obesity who did not identify significant improvements in SMMI in favor of ST (*p* = 0.38). It is important to mention that the meta-analysis by Yang, Ye, Zhu, Zhang, Liu, Xie, Long, Huang, Niu, Luo and Wang [17] only included two studies, unlike our meta-analysis, which included four studies and five experimental groups. The studies analyzed in the present meta-analysis by ST used intensities for resistance training at 50% 1 RM [35], 60% at 85% 1 RM [36,39], and EBT [38]. In this sense, progressive ST effectively improves muscle strength and increases muscle mass in older people [42,43], since it can promote protein synthesis and activate key molecular signaling pathways (PI3K-Akt-mTOR) that regulate muscle fiber metabolism and function [44]. However, the combination of ST and protein supplementation may be important for optimal muscle mass maintenance and recovery in older people [44]. This may be reflected in our meta-analysis, specifically in the results of the previous studies [36,39] that prescribed progressive resistance training with intensities of 60% to 85% 1 RM along with a daily protein intake of 1.5–1.6 g/kg/d, obtaining the largest ES (2.7 and 2.4, respectively). In this context, protein supplementation may enhance the effect of ST on skeletal muscle signaling by increasing anabolic pathways such as mTORc1 and decreasing catabolic pathways in the body [44,45]. This was confirmed through sensitivity and influence analyses. Specifically, the exclusion of the studies by Kemmler, Schoene, Kohl and von Stengel [37] and Lee, Lee, Lin, Liao, Liou and Huang [38] yielded a statistically significant effect in favor of ST along with a reduction in heterogeneity. These findings suggest that, although these studies partially amplified the overall effect, improvements on SMMI in older people with osteosarcopenia remain significant even after excluding studies with extreme estimates or potential bias, thus confirming the robustness of the meta-analysis effect.

#### 4.1.3. Bone Mineral Density

No significant improvements were found for BMD in favor of ST. This is in contrast to a meta-analysis by Yang, Ye, Zhu, Zhang, Liu, Xie, Long, Huang, Niu, Luo and Wang [17] in older people with osteosarcopenic obesity who reported significant improvements in BMD in favor of ST (*p* = 0.03). This is similar to the findings of a recent meta-analysis by Liu and Lee [16] in older females with osteosarcopenic obesity who reported significant improvements in BMD in favor of ST (*p* < 0.001). The discrepancy between studies may be explained by differences in population characteristics, training variables, and follow-up duration. It is well established that loss of muscle and bone mass is a hallmark of aging [45], and both tissues are interconnected through mechanical and endocrine pathways that are highly responsive to physical activity [46,47]. In particular, regular loading exercise promotes osteogenesis and muscle anabolism via growth factors and myokines such as insulin-like growth factor-1 and interleukin-6, which regulate protein synthesis and bone turnover [46,47,48]. Although our meta-analysis did not demonstrate significant improvements, a trend toward benefit was evident, suggesting that longer interventions or higher-intensity mechanical stimuli may be required to induce measurable gains in BMD. Indeed, bone adaptations typically demand prolonged exposure to high strain rates and dynamic loads to stimulate remodeling [46,47,48]. Importantly, maintaining BMD is itself clinically relevant, as preventing bone loss contributes to reducing fracture risk and preserving functional independence [49]. Supporting this, studies that did not observe increases in BMD after ST nonetheless reported functional gains, such as better performance in the timed up-and-go test, enhanced endurance, and lower fall risk [48,50,51,52]. These functional improvements are consistent with the significant gains in MIHS and the trend toward faster gait speed observed in our analysis, reinforcing the idea that muscular and neuromotor adaptations may precede detectable skeletal changes. Finally, sensitivity analysis indicated that the exclusion of studies with a high risk of bias, i.e., Banitalebi, Ghahfarrokhi and Dehghan [34] and Banitalebi, Faramarzi, Ghahfarokhi, SavariNikoo, Soltani and Bahramzadeh [33], reduced the estimated magnitude of benefit but did not change the overall direction of the effect. This suggests that, while the pooled results were partially influenced by methodological limitations, the conclusion remains robust: ST alone may not consistently increase BMD in the short term, but it can stabilize bone mass and generate functional benefits that are equally relevant in the clinical management of osteosarcopenia.

### 4.2. Physical Performance

#### 4.2.1. Maximum Isometric Handgrip Strength

Significant improvements were found for MIHS in favor of ST. This is in line with that reported in a systematic review by Atlihan, et al. [53] in patients with osteosarcopenia, reporting significant improvements in MIHS in favor of ST (*p* < 0.001), and is similar to the findings of Tsai, et al. [54] in a meta-analysis of older people with sarcopenia, reporting significant increases in MIHS in favor of ST using elastic bands (*p* = 0.002) compared to active/inactive control groups. Likewise, Zhao, et al. [55], in a meta-analysis of older people with sarcopenia, found significant increases in MIHS in favor of ST using EBT (*p* < 0.05) compared to inactive control groups. This is the first systematic review to meta-analyze the MIHS variable in older people with osteosarcopenia. Handgrip strength is useful as an indicator of muscle strength, where low MIHS values have been associated with sarcopenia [56], low BMD, and increased risk of osteoporosis and fractures [57]. Furthermore, BMD obtained by dual X-ray absorptiometry is related to MIHS [58]. In this context, its assessment and improvement through training interventions becomes clinically relevant [56]. Our meta-analysis for MIHS included three studies [33,38,39]. Two performed an ST program using EBT [33,38] and one performed an ST program using machines and dumbbells [39]. All three interventions performed full-body multi-joint exercises at intensities of 13 RPE on the Borg scale and at 70% to 85% of 1 RM. In this sense, ST using elastic bands implies that, as the range of motion increases, the resistance generated by the elastic band is greater, forcing subjects to maintain muscle power at higher overload ranges [59]. Furthermore, the upper body exercises involved constant gripping and pulling, which may explain the increases in MIHS. On the other hand, the increase in MIHS using machines and free weights, similar to EBT, can be explained by the execution of exercises focused on the upper body, specifically on the biceps, triceps, and deltoids through flexion and extension movements, which may have contributed to the increase in forearm strength and, consequently, MIHS [60]. Alternatively, sensitivity analysis could not be conducted due to the limited number of studies included in the meta-analysis (n = 3). Therefore, the results for this outcome should be interpreted with caution, taking into account this methodological limitation.

#### 4.2.2. Gait Speed

No significant improvements were found for gait speed in favor of ST. This is similar to the findings in a systematic review with meta-analysis by Yang, Ye, Zhu, Zhang, Liu, Xie, Long, Huang, Niu, Luo and Wang [17] in older people with osteosarcopenic obesity who did not report significant improvements in gait speed in favor of ST, although this variable was not meta-analyzed due to lack of data for the analysis [17]. Likewise, a systematic review by Atlihan, Kirk and Duque [53] in patients with osteosarcopenia did not identify significant improvements for gait speed in favor of high-intensity strength training interventions. The above confirms the findings of a recent systematic review with meta-analysis by Liu and Lee [16] in older females with osteosarcopenic obesity, where no significant improvements in gait speed in favor of ST were reported. These authors highlighted that the results were inconclusive due to inconsistent findings and limited data for a meta-analysis. From a functional perspective, gait speed is often considered a surrogate marker of lower limb muscle strength and overall mobility [39]. However, its regulation is multifactorial, and improvements cannot be attributed solely to gains in muscle mass or strength. As highlighted by Lichtenberg, von Stengel, Sieber and Kemmler [39], gait speed may also be influenced by age-related motor neuron degeneration, joint range of motion restrictions, and non-muscular factors such as balance control, cognitive status, or depressive symptoms. These additional determinants may limit the capacity of ST to elicit measurable improvements in gait speed, even when muscle function is enhanced. Clinically, this implies that, while ST remains essential to counteract sarcopenia and support musculoskeletal health, it may need to be combined with other modalities—such as balance training, mobility drills, or dual-task cognitive exercises—to effectively improve gait speed in older people with osteosarcopenia. The limited number of trials available for this outcome underscores the need for further high-quality research that integrates multimodal interventions. Until then, findings regarding gait speed should be interpreted cautiously, acknowledging both methodological limitations and the complex interplay of factors beyond muscle strength that influence walking performance.

### 4.3. Macro- and Micronutrient Intake

#### 4.3.1. Protein Intake

Our meta-analysis reported significant improvements in protein intake in favor of ST. Three of the meta-analyzed studies prescribed whey protein supplementation between 1.5 and 1.6 g/kg/d [36,39]. This is in line with the recommendations of a systematic review with meta-analysis by Gielen, Beckwée, Delaere, De Breucker, Vandewoude, Bautmans, Gerontology and Geriatrics [45], on nutritional interventions to improve muscle mass, muscle strength, and physical performance in older people who suggested up to >1.2 to 1.5 g/kg of body weight for older people with an acute or chronic disease. Furthermore, all studies followed up with questionnaires from the beginning to the end of the interventions to monitor individual protein intake in participants. Our findings demonstrate that older people can sustain and accept protein supplementation when prescribed and monitored weekly by telephone calls and quick and easy questionnaires. In this context, dietary intake can be considered of great relevance in older people since a reduction in protein intake is closely associated with skeletal muscle loss [45]. Furthermore, it has been reported that poor protein intake is significantly related to osteosarcopenia in older people compared to sarcopenic obesity [61]. Similarly, it has been reported that a high protein intake may have a protective role in BMD in the lumbar spine, compared to low protein intake [62]. It is important to distinguish protein quality and timing to maximize the effect of protein supplementation in conjunction with ST. For example, it is well documented that “fast” proteins (e.g., whey protein) can stimulate protein synthesis to a greater extent than “slow” proteins (e.g., casein, another milk-derived protein) [63]. Furthermore, evenly distributed protein intake throughout the day, with 25–30 g per meal, has been suggested to optimize protein synthesis in older people [45]. Regarding the sensitivity and influence analysis, the exclusion of the study by Banitalebi, Faramarzi, Ghahfarokhi, SavariNikoo, Soltani and Bahramzadeh [33] resulted in a substantial reduction in heterogeneity while maintaining a statistically significant overall effect. These findings highlight that the inclusion of Banitalebi, Faramarzi, Ghahfarokhi, SavariNikoo, Soltani and Bahramzadeh [33] artificially inflated the magnitude of the effect and that its exclusion allows for a more conservative and homogeneous interpretation of the impact of ST on protein intake in older people with osteosarcopenia.

#### 4.3.2. Calcium Intake

No significant improvements were found for calcium intake in favor of ST. Two meta-analyzed studies [36,39] provided a calcium intake of approximately 1000 mg/day. This aligns with current recommendations of 1000 to 1200 mg/day in older people [64,65]. As with protein intake, all studies followed up with questionnaires from the beginning to the end of the interventions to control the individual calcium intake of the participants. The fact that our meta-analysis did not identify significant increases in calcium intake in favor of ST may be attributed to the fact that two of the meta-analyzed studies prescribed the same dose of calcium (approximately 1000 mg/day) in the experimental and control groups [36,39]. In this regard, calcium intake remained stable during the interventions from the beginning to the end. Calcium has been recognized as the great “bone nutrient”, since almost 99% of the calcium in the adult human body is contained in the bones as hydroxyapatite [66]. Milk and its derivatives are foods with an optimal source of calcium and have an important role in bone health. The mechanism by which calcium intake affects bone health is by increasing BMD [10]. Tai, Leung, Grey, Reid and Bolland [10], in a meta-analysis on calcium intake and BMD in older people, reported that increasing calcium intake from dietary sources slightly increased BMD (by 0.6–1.8%) for 1 to 2 years in the lumbar spine, total hip, femoral neck, and total body. However, increases in BMD were small and nonprogressive, with no reduction in rates of BMD loss over 1 year. On the other hand, Tai, Leung, Grey, Reid and Bolland [10] reported in their subgroup meta-analysis that there were no differences in changes in BMD with calcium doses of ≥1000 mg/day and <1000 mg/day or doses of ≤500 mg/day and >500 mg/day. This suggests that increasing calcium intake, whether from dietary sources or calcium supplements, provides a slight, nonprogressive increase in BMD, with no continued reduction in rates of BMD loss beyond 1 year [10]. Based on current evidence, minor effects on BMD are unlikely to translate into clinically meaningful reductions in fracture risk in older people. Moreover, it is important to note that sensitivity analysis could not be performed due to the limited number of studies included in the meta-analysis (n = 3). This methodological constraint further limits the certainty of the findings, and thus, the results related to calcium intake should be interpreted with caution until more robust evidence becomes available.

### 4.4. Subgroup Analysis by Type ST

#### 4.4.1. Bone Mineral Density

No significant differences in BMD were found for resistance training and EBT, unlike what was reported in the previous meta-analysis [16,17] in older people with osteosarcopenic obesity who reported significant improvements in BMD in favor of ST. However, neither meta-analysis performed a subgroup analysis to identify possible differences in BMD for resistance training and EBT. O’Bryan, Giuliano, Woessner, Vogrin, Smith, Duque and Levinger [48], in a meta-analysis that aimed to quantify concomitant changes in lower body muscle strength and BMD in older people following an ST program, reported significant increases in the subgroup analysis for femur/hip and lumbar spine BMD in favor of resistance training and combined resistance training plus weight-bearing/impact-loading exercises with no differences between them. The reasons for the discrepancy in our findings may be attributed to the high risk of bias in two of the meta-analyzed studies for EBT [33,34] and the moderate intensity (RPE 13 Borg scale and 50% 1 RM) used in four of the studies [33,34,35,38]. It has been suggested that high external loads (e.g., 75–80% 1 RM) may be better for inducing favorable changes in BMD in middle-aged and older people [48]. Furthermore, it has been reported that bone requires a more extended time and higher dynamic strain rates to generate positive adaptations following training [46,48]. In this sense, increased mechanical loads stimulate bone modeling and remodeling to increase bone mass and stiffness [46,48]. Finally, BMD results may be influenced by nutritional factors not controlled for in the overall studies, such as individual food and nutrient intake (i.e., daily calories, carbohydrate, fat, protein, vitamin D, calcium, and phosphate) [64]. Moreover, due to the limited number of studies available for this outcome (n = 3), a sensitivity analysis could not be conducted. This methodological constraint limits the ability to assess the robustness of the pooled estimate, and therefore, the findings regarding BMD should be interpreted with caution.

#### 4.4.2. Body Fat Percentage

Significant differences were found in BFP in favor of EBT without finding significant differences in resistance training. It has been reported that ST, through its different modalities (i.e., elastic bands, free weights, and machines), can improve body composition in older people with sarcopenia [42]. The discrepancies in the literature can be attributed to the morphological characteristics of the participants, nutritional factors, and training variables such as the interventions’ duration, frequency, and intensity. Regarding our results, when analyzing the characteristics of the participants in the EBT subgroup, we identified that the experimental groups were characterized by being osteosarcopenic older people with obesity and adiposity [33,34], unlike the participants in the resistance training subgroup, who were characterized as mainly older people with osteosarcopenia conditions and without obesity [39]. For example, in previous studies [33,34], the control group and the experimental group started the interventions with a BFP of 43.60 ± 2.66 and 46.29 ± 3.42, respectively. This is unlike the study by Kemmler, Schoene, Kohl and von Stengel [37], where the control and experimental groups presented a BFP before the intervention of 34.2 ± 6.1 and 33.6 ± 4.0, respectively. Higher initial BFP offers greater potential for morphological improvement when exposed to adequate training stimuli Buskard and Petrella [42]. Additionally, EBT interventions in the included studies were performed three times per week, targeting large muscle groups with moderate RPE (Borg 10–13), a configuration that can increase total energy expenditure and favor fat loss. In contrast, some resistance training protocols involved lower training volumes (e.g., one set per exercise in Cunha, Ribeiro, Tomeleri, Schoenfeld, Silva, Souza, Nascimento, Sardinha and Cyrino [35]) or participants with lower initial adiposity, potentially limiting the magnitude of change. It is also important to consider that differences in dietary intake monitoring across studies, e.g., De Rui, Inelmen, Pigozzo, Trevisan, Manzato and Sergi [64], may have further influenced the observed between-modality discrepancies. Together, these factors likely contributed to the superior reduction in BFP observed in the EBT subgroup. It is also important to note that a sensitivity analysis could not be performed due to the small number of studies available for this outcome (n = 3). As such, the current findings regarding BFP should be interpreted with caution, given the limited ability to explore the stability of the ES.

Regarding the certainty of evidence, our meta-analysis rated it as moderate to low, which limits the possibility of issuing definitive recommendations on the use of ST to improve body composition, physical performance, and protein or calcium intake in older people with osteosarcopenia. These findings are consistent with the systematic review by Atlihan, Kirk and Duque [53], who also reported moderate to low certainty of evidence when analyzing non-pharmacological interventions for body composition in 106 older people with osteosarcopenia. Likewise, a network meta-analysis by Shen, et al. [67], which examined the effects of ST, aerobic exercise, and their combination on physical performance in 3728 participants with sarcopenia, reported only moderate certainty of evidence. This pattern across reviews highlights common limitations, including heterogeneity in sample size and participant characteristics, methodological quality, and the risk of bias of the included studies, as well as differences between experimental and control groups. Together, these factors restrict the strength of current conclusions and reinforce the need for future trials with larger, more homogeneous populations and more rigorous study designs.

### 4.5. Limitations

This study also presents several limitations that should be considered when interpreting its findings. The limited number of studies included in the meta-analysis may restrict the statistical power and generalizability of the results, suggesting that including more studies could provide a more comprehensive and precise estimate of the effects of ST on osteosarcopenia. The moderate to high heterogeneity of the included studies does not allow definitive conclusions to be drawn about some of the variables analyzed, which may be due to the low sample size. Additionally, the risk of bias in several included studies raises concerns about the reliability of the results, indicating that addressing these biases in future research could strengthen the evidence base for ST interventions in this population. Moreover, although sensitivity and influence analyses were feasible for some outcomes (BFP, SMMI, BMD, and protein intake), the results were often highly sensitive to individual studies—particularly those with a high risk of bias—highlighting the fragility of some effect estimates and reinforcing the need for cautious interpretation. The short intervention time in many of the included studies may not be sufficient to observe the long-term effects of ST on BMD and other outcomes, underscoring the need for future studies to consider more extended intervention periods to assess the sustained benefits of ST. Furthermore, the lack of subgroup analysis based on the presence of obesity may influence the analysis of morphological variables such as BFP, suggesting that stratifying the analysis by obesity status could provide a more nuanced understanding of the effects of ST on body composition. Finally, the absence of a training volume subgroup analysis (e.g., <3 sets vs. >3 sets) limits the ability to optimize exercise prescriptions for older people with osteosarcopenia, highlighting the potential benefits of analyzing the impact of different training volumes.

### 4.6. Strengths

This study presents several notable strengths. Its comprehensive meta-analysis integrates data from multiple RCTs, providing a robust assessment of the effects of ST on various outcomes in older people with osteosarcopenia. By incorporating a diverse range of studies, this approach enhances statistical power and improves the generalizability of the findings. Methodological rigor is ensured through adherence to the PRISMA statement and the use of established assessment tools such as TESTEX, RoB 2, and GRADE, reinforcing the reliability and validity of the conclusions. Additionally, including subgroup analyses based on the type of ST (EBT vs. resistance training) offers valuable insights into the differential effects of these interventions on body composition, aiding in identifying the most effective ST modalities for specific outcomes. Finally, this study’s consideration of nutritional factors examines the impact of ST on macro- and micronutrient intake, highlighting the importance of protein supplementation in conjunction with physical activity, reflecting a holistic approach that recognizes the interplay between physical activity and nutrition in managing osteosarcopenia.

### 4.7. Practical Applications

Exercise Program Design: this study highlights the effectiveness of ST in improving SMMI, MIHS, and protein intake in older people with osteosarcopenia. Application: Clinicians and exercise professionals can design targeted ST programs that include physical activity focusing on both upper and lower body muscle strength. To maximize benefits, these programs should consider incorporating EBT and resistance training. Example: a practical physical activity routine could involve squats, lunges, bicep curls, triceps extensions, and rows, performed 2–3 times per week with adequate rest days.

Nutritional Guidance: this meta-analysis indicates significant improvements in protein intake among participants undergoing ST. Application: Healthcare providers can recommend and monitor protein supplementation, particularly whey protein, in older people undergoing ST. A protein intake of 1.5–1.6 g/kg/day is shown to be effective. Example: Nutritional plans should include protein-rich foods such as lean meats, dairy products, legumes, and nuts. Whey protein supplements can meet the recommended daily intake, especially for those who struggle to consume enough protein through diet alone.

EBT (elastic band training): this study reveals that EBT is particularly effective in reducing BFP in older people with osteosarcopenia. Application: Incorporating EBT into exercise regimens can be a cost-effective and accessible way to manage body composition in this population. EBT is especially useful for individuals with limited access to traditional gym equipment. Example: Simple EBT exercises can include seated rows, bicep curls, lateral walks, and leg extensions. These exercises can be easily performed at home with minimal equipment.

### 4.8. Clinical Applications

Assessment and Diagnosis: this study underscores the importance of assessing body composition, muscle strength, and physical performance in older people to diagnose osteosarcopenia early. Application: clinicians should routinely screen older patients for signs of muscle and bone loss using tools such as DXA scans for BMD and MIHS measurements for muscle strength. Example: regular geriatric assessments should include assessments of gait speed, chair stand tests, and MIHS to identify individuals at risk of osteosarcopenia.

Intervention Strategies: this research supports using ST as a non-pharmacological intervention to improve muscle mass and strength in older people with osteosarcopenia. Application: Clinicians can prescribe ST programs as part of a comprehensive treatment plan for managing osteosarcopenia. These programs should be tailored to the individual’s physical capabilities and health status. Example: a clinical intervention might involve a combination of supervised ST sessions and home-based exercises, along with nutritional counseling to ensure adequate protein and calcium intake.

Monitoring Treatment Outcomes: this study highlights specific metrics such as SMMI, MIHS, and BFP as key indicators of treatment effectiveness. Application: clinicians should regularly monitor these parameters to assess the impact of ST interventions and make necessary adjustments to optimize patient outcomes. Example: follow-up assessments should include periodic DXA scans to track changes in BMD, SMMI, and MIHS measurements to monitor improvements in muscle function.

### 4.9. Epidemiological Applications

Risk Factor Identification: this study emphasizes the aging process as a significant risk factor for osteosarcopenia, leading to decreased body composition and increased risk of geriatric syndromes. Application: Public health initiatives can target older people with screening programs to identify individuals at high risk of developing osteosarcopenia. Early detection allows for timely interventions to mitigate disease progression. Example: Population-based studies can assess the prevalence of osteosarcopenia in different age groups and identify modifiable risk factors such as physical inactivity and poor nutrition.

Intervention Effectiveness: this meta-analysis provides evidence supporting the effectiveness of ST in improving outcomes related to osteosarcopenia. Application: Public health campaigns can promote ST as a preventive and therapeutic strategy for managing osteosarcopenia in older people. These campaigns should highlight the benefits of ST in improving muscle mass, strength, and overall physical function. Example: community-based physical activity programs can be implemented to provide older people access to supervised ST sessions and promote adherence to regular physical activity.

Healthcare Resource Allocation: this study highlights the high healthcare expenditures associated with osteosarcopenia, emphasizing the need for cost-effective interventions. Application: Healthcare policymakers can allocate resources toward promoting ST and nutritional interventions to reduce the burden of osteosarcopenia on healthcare systems. Investing in preventive strategies can lead to long-term cost savings by reducing the incidence of falls, fractures, and other complications associated with the condition. Example: economic evaluations can assess the cost-effectiveness of different ST programs and inform decisions regarding resource allocation for geriatric care.

## 5. Conclusions

ST in older people with osteosarcopenia leads to increased SMMI, MIHS, and protein intake. EBT is effective in decreasing BFP compared to traditional resistance training. This type of training is economical and has a solid clinical impact on older people with osteosarcopenia worldwide. This provides new strategies to combat this geriatric syndrome, which is associated with various complications in the general health status of older people, affecting their quality of life.

## Figures and Tables

**Table 1:** Selection criteria used in the systematic review with meta-analysis.

Category	Inclusion Criteria	Exclusion Criteria
Population	Participants were required to have a combination of osteopenia and sarcopenia and no other diseases, such as fractures, heart failure, or diabetes. People were mean ≥60 years and over, but there were no restrictions by sex or setting (such as hospitals, communities, or nursing homes).	Population under 60 years of age with osteoporosis or sarcopenic obesity.
Intervention	Interventions included ST, such as EBT and progressive ST, for 4 weeks or more.	Interventions that do not use ST. There are no details of the intervention procedure.
Comparator	Interventions with or without an active/inactive control group or placebo.	Lack of baseline and/or follow-up data. Absence of control group.
Outcome	Primary outcomes: body composition (e.g., BFP, SMMI, BMD). Secondary outcomes: physical performance (e.g., MIHS and gait speed) and intake of protein and/or calcium.	Lack of baseline data and/or follow-ups.
Study design	Experimental design studies (randomized controlled trials) with pre- and post-assessments.	Non-randomized controlled trials, cross-sectional, retrospective, and prospective studies.

ST: strength training. EBT: elastic band training. BFP: body fat percentage. SMMI: skeletal muscle mass index. BMD: bone mineral density. MIHS: maximal isometric handgrip strength.

**Table 2:** Study quality assessment according to the TESTEX scale.

Study	Eligibility Criteria Specified	Randomly Allocated Participants	Allocation Concealed	Groups Similar at Baseline	Assessors Blinded	Outcome Measures Assessed >85% of Participants *	Intention to Treat Analysis	Reporting of between Group Statistical Comparisons	Point Measures and Measures of Variability Reported **	Activity Monitoring in Control Group	Relative Exercise Intensity Reviewed	Exercise Volume and Energy Expended	Overall TESTEX #
Banitalebi, Faramarzi, Ghahfarokhi, SavariNikoo, Soltani and Bahramzadeh [33]	Yes	Yes	Yes	Yes	Yes	Yes (2)	Yes	Yes	Yes (1)	Yes	Yes	Yes	15/15
Banitalebi, Ghahfarrokhi and Dehghan [34]	Yes	Yes	Yes	Yes	No	Yes (2)	Yes	Yes	Yes (1)	Yes	Yes	Yes	14/15
Cunha, Ribeiro, Tomeleri, Schoenfeld, Silva, Souza, Nascimento, Sardinha and Cyrino [35]	Yes	Yes	Yes	Yes	Yes	Yes (1)	No	Yes	Yes (1)	Yes	Yes	Yes	13/15
Kemmler, Kohl, Fröhlich, Schoene and von Stengel [36]	Yes	Yes	Yes	Yes	Yes	Yes (1)	Yes	Yes	Yes (1)	Yes	Yes	Yes	14/15
Kemmler, Schoene, Kohl and von Stengel [37]	Yes	Yes	Yes	Yes	Yes	Yes (1)	Yes	Yes	Yes (1)	Yes	Yes	Yes	14/15
Lee, Lee, Lin, Liao, Liou and Huang [38]	Yes	Yes	Yes	Yes	Yes	Yes (1)	Yes	Yes	Yes (1)	Yes	Yes	Yes	14/15
Lichtenberg, von Stengel, Sieber and Kemmler [39]	Yes	Yes	Yes	Yes	Yes	Yes (1)	Yes	Yes	Yes (1)	Yes	Yes	Yes	14/15

* Three points are possible: one point if adherence >85%, one point if adverse events were reported, and one point if exercise attendance was reported. ** Two points possible: one point if the primary outcome is reported and one point if all other outcomes were reported. # total out of 15 points. TESTEX: tool for assessing study quality and reporting in exercise.

**Table 3:** Characteristics of participants examined in the included studies.

Authors	Study Design	Country	Sex and Age	Groups (Sample Size)	Body Composition Assessment Tool	Diagnostic Criteria for Osteosarcopenia	Training			Intensity and Color Band	Primary Outcome	Secondary Outcome	Adverse Events	Adherence
							Weeks	Frequency (Sessions/ Weeks)	Minutes					
Banitalebi, Faramarzi, Ghahfarokhi, SavariNikoo, Soltani and Bahramzadeh [33]	RCT	Iran	100% Female ST: 64.11 ± 3.81 CG: 64.05 ± 3.35	ST (32): elastic band training CG (31): continued with their daily life activities	DXA	−2.5 ≤ T-score ≤ −1.0 of L1–L4, and/or total femur or femoral neck, BFP > 32%, body mass index (BMI) > 30 kg/m^2^, −2.5 ≤ T-score ≤ −1.0 of L1–L4, and/or total femur (TF) or femoral neck (FN), gait speed (10 m walk test (10 MWT)) ≤ 1 (m/s), and skeletal muscle mass index (SMI) ≤ 28% or ≤7.76 kg/m^2^	12	3	60	OMNI-RES (10–13) (Thera-Band^®^, The Hygienic Corporation, Akron, OH, USA; yellow color progressing to red and ending in black).	Body fat (%) BMD (gr/cm^2^)	MIHS (kg) TUG (s) Gait speed (m/s) 30-second chair stand (rep) Protein intake (g/kg/d) Calcium intake (g/kg/d)	Not observed	>85%
Banitalebi, Ghahfarrokhi and Dehghan [34]	RCT	Iran	100% Female ST: 64.11 ± 3.81 CG: 64.05 ± 3.35	ST (32): elastic band training CG (31): continued with their daily life activities	DXA	−2.5 ≤ T-score ≤ −1.0 of L1–L4, and/or total femur or femoral neck, BFP > 32%, body mass index (BMI) > 30 kg/m^2^, −2.5 ≤ T-score ≤ −1.0 of L1–L4, and/or total femur (TF) or femoral neck (FN), gait speed (10 m walk test (10 MWT)) ≤ 1 (m/s), and skeletal muscle mass index (SMI) ≤ 28% or ≤7.76 kg/m^2^	12	3	60	OMNI-RES (10–13) (Thera-Band^®^, The Hygienic Corporation, Akron, OH, USA; yellow color progressing to red and ending in black).	Body fat (%) BMD (gr/cm^2^) Bone mass content (gr)		Not observed	>85%
Cunha, Ribeiro, Tomeleri, Schoenfeld, Silva, Souza, Nascimento, Sardinha and Cyrino [35]	RCT	Brazil	100% Female ST1: 66.6 ± 5.1 ST2: 68.3 ± 4.2 CG: 67.3 ± 3.6	ST1 (23): Resistance training ST2 (23): Resistance training CG (23): continued with their daily life activities	DXA	Z-score, derived from the average of the components, was calculated using the following formula: (muscular strength Z-core) + (SMM Z-score) + (−1 x body fat Z-score) + (BMD Z-score)/4	12	ST1: 1 ST2: 3	60	50% 1 RM	Body fat (%) BMD (gr/cm^2^) SMMI (kg/m^2^)	No reported	No reported	>85%
Kemmler, Kohl, Fröhlich, Schoene and von Stengel [36]	RCT	Germany	100% Male ST: 77.8 ± 3.6 CG: 79.2 ± 4.7	ST (21): Hit Resistance training CG (21): continued with their daily life activities	DXA, BIA	−2.5 ≤ T-score ≤ −1.0 of L1–L4, and/or total femur or femoral neck, BFP > 32%, body mass index (BMI) > 30 kg/m^2^, −2.5 ≤ T-score ≤ −1.0 of L1–L4, and/or total femur (TF) or femoral neck (FN), gait speed (10 m walk test (10 MWT)) ≤ 1 (m/s), and skeletal muscle mass index (SMI) ≤ 28% or ≤7.76 kg/m^2^	18 months	3	60	85% 1 RM	SMMI (kg/m^2^)	Protein intake (g/kg/d) Calcium intake (g/kg/d)	No reported	>85%
Kemmler, Schoene, Kohl and von Stengel [37]	RCT	Germany	100% Male ST: 77.8 ± 3.6 CG: 79.2 ± 4.7	ST (21): Hit Resistance training CG (21): continued with their daily life activities	DXA, BIA	−2.5 ≤ T-score ≤ −1.0 of L1–L4, and/or total femur or femoral neck, BFP > 32%, body mass index (BMI) > 30 kg/m^2^, −2.5 ≤ T-score ≤ −1.0 of L1–L4, and/or total femur (TF) or femoral neck (FN), gait speed (10 m walk test (10 MWT)) ≤ 1 (m/s), and skeletal muscle mass index (SMI) ≤ 28% or ≤7.76 kg/m^2^	18 months	3	60	60% to 85%	Body fat (%)	Protein intake (g/kg/d) Calcium intake (g/kg/d)	No reported	>85%
Lee, Lee, Lin, Liao, Liou and Huang [38]	RCT	Taiwan, China	100% Female ST: 70.13 ± 4.41 CG: 71.82 ± 5.23	ST (15): elastic band training CG (12): continued with their daily life activities	DXA, BIA	European Working Group on Sarcopenia in Older People (EWGSOP) of low muscle mass (less than 5.67 kg/m^2^ based on DXA) and a grip strength of <20 kg or gait speed (GS) of <0.8 m/s was diagnosed with sarcopenia. Osteopenia was diagnosed according to the aforementioned WHO criteria (T-score < 1.0). We measured BMD by using the standard protocol. Lumbar spine BMD was determined through the imaging of lumbar vertebra one through four (L1–L4), which included the body of the vertebra, the pedicles, lamina, spinous process, and transverse processes.	12	3	NR	13 RPE Borg (Thera-Band^®^, The Hygienic Corporation, Akron, OH, USA; yellow color progressing to red, green, and blue and ending in black or silver).	Body fat (%) BMD (gr/cm^2^) SMMI (kg/m^2^)	MIHS (kg) TUG (s) Gait speed (m/s) 30-second chair stand (rep)	Not observed	>85%
Lichtenberg, von Stengel, Sieber and Kemmler [39]	RCT	Italy	100% Male ST: 77.8 ± 3.6 CG: 79.2 ± 4.7	ST (21): Hit Resistance training CG (22): continued with their daily life activities	DXA	(a) SMI, as assessed by DXA, was below 7.26 kg/m^2^ (≤−2 standard deviations (SD) T-score, i.e., sarcopenia), (b) bone mineral density at the region of interest (ROI), i.e., either the lumbar spine or the proximal femur (total hip or femoral neck) was ≤−1 SD T-score (i.e., osteopenia),	12	NR	NR	70% to 85% 1 RM	SMMI (kg/m^2^)	MIHS (kg) TUG (s) Gait speed (m/s) Protein intake (g/kg/d)	Not observed	>85%

RCT: randomized controlled trials; ST: strength training; CG: control group; DXA: dual X-ray absorptiometry; BMD: bone mineral density; SMMI: skeletal muscle mass index; NR: not reported; 1 RM: one-repetition maximum; TUG: timed up-and-go; MIHS: maximal isometric handgrip strength; KG: kilograms; S: seconds; m/s: meters per seconds; rep: repetitions; %: percentages; kg/m^2^: kilograms per square meter.

**Table 4:** Synthesis of the studies’ results, including the effects of strength training on body composition, physical performance, and protein or calcium intake in older people with osteosarcopenia.

	n ^a^	Model of Effect	ES (95%CI)	*p*	I^2^ (%)	Egger’s Test (*p*)	RW (%)
**Body composition**							
Body fat percentage	5,6,5,	Random	0.64 (−0.12 to 1.41)	0.10	89.5	0.00	4.18–7.61
SMMI (kg/m^2^)	4,5,4,	Random	1.48 (0.61 to 2.35)	0.001	85.2	0.00	2.51–6.37
BMD (gr/cm^2^)	5,6,5,	Fixed	0.22 (−0.01 to 0.46)	0.06	0.00	0.84	4.92–12.6
**Physical performance**							
MIHS both hands (kg)	3,3,3	Random	1.38 (0.10 to 2.65)	0.03	89.9	0.00	3.26–6.52
Gait speed (m/s)	3,3,3	Fixed	0.33 (−0.01 to 0.67)	0.06	0.00	0.86	2.51–4.11
**Macro- and micronutrient intake**							
Protein intake (g/kg/d)	4,4,4	Random	0.91 (0.05 to 1.78)	0.03	87.8	0.00	4.68–7.33
Calcium intake (g/kg/d)	3,3,3	Random	0.20 (−0.10 to 0.52)	0.19	83.1	0.00	3.67–6.61

Note: Bolded *p*-values mean significant improvement (*p* < 0.05) in the experimental group after the strength training compared to the control group, and *p* > 0.05 represents a low risk of publication bias. ^a^ Data indicate the number of studies that provided data for analysis, the number of experimental and control groups, and the total number of female soccer players included in the analysis. Abbreviations: BMD: bone mineral density; SMMI: skeletal muscle mass index; 95%CI: 95% confidence interval; ES: effect sizes (Hedge’s g); RW: relative weight of each study in the analysis.

**Table 5:** Comparative effects of elastic band training (EBT) and resistance training (RT) on body composition, physical performance, and protein or calcium intake outcomes in older people with osteosarcopenia.

Outcome	EBT–Effect (*p*)	RT–Effect (*p*)	Comparative Interpretation
Body fat percentage (BFP)	↓ Significant (*p* = 0.01)	NS (*p* = 0.76)	EBT more effective in reducing BFP
Skeletal muscle mass index (SMMI)	↑ Significant (*p* < 0.01)	↑ Significant (*p* < 0.01)	Both modalities effective
Bone mineral density (BMD)	NS (*p* = 0.16)	NS (*p* = 0.11)	No significant differences
Maximal isometric handgrip strength (MIHS)	↑ Significant (*p* < 0.05)	↑ Significant (*p* < 0.05)	Both modalities effective
Gait speed	NS	NS	No differences
Protein intake	↑ Significant (*p* < 0.05)	↑ Significant (*p* < 0.05)	Both modalities effective
Calcium intake	NS	NS	No differences

Abbreviations: NS = not significant; ↑ = significant increase; ↓ = significant decrease.

**Table 6:** GRADE assessment for the certainty of evidence.

Assessment of Certainty							Number of Patients		Effect		Certainty	Importance
Number of Studies	Study Design	Risk of Bias	Inconsistency	Indirect Evidence	Vagueness	Other Considerations	Strength Training	Active Control Group	Relative (95% CI)	Absolute (95% CI)		
Osteosarcopenic obesity markers following elastic band resistance training: TA randomized controlled trial												
1	randomized trials	very serious ^a^	not serious	not serious	not serious	none	32/63 (50.8%)	31/63 (49.2%)	not estimable		⨁⨁ ◯◯ Go down ^a^	IMPORTANT
Effect of 12-week elastic band resistance training on MyomiRs and osteoporosis markers in elderly women with Osteosarcopenic obesity: a randomized controlled trial												
1	randomized trials	very serious ^a^	not serious	not serious	not serious	none	32/63 (50.8%)	31/63 (49.2%)	not estimable		⨁⨁ ◯◯ Go down ^a^	IMPORTANT
The effects of resistance training volume on osteosarcopenic obesity in older women												
1	randomized trials	serious ^b^	not serious	not serious	not serious	none	41/62 (66.1%)	21/62 (33.9%)	not estimable		⨁⨁⨁ ◯ Moderate ^b^	IMPORTANT
Detraining effects after 18 months of high-intensity resistance training on osteosarcopenia in older men—Six-month follow-up of the randomized controlled Franconian Osteopenia and Sarcopenia Trial (FrOST)												
1	randomized trials	serious ^b^	not serious	not serious	not serious	none	21/43 (48.8%)	22/43 (51.2%)	not estimable		⨁⨁⨁ ◯ Moderate ^b^	IMPORTANT
Changes in Body Composition and Cardiometabolic Health After Detraining in Older Men with Osteosarcopenia: 6-Month Follow-Up of the Randomized Controlled Franconian Osteopenia and Sarcopenia Trial (FrOST) Study												
1	randomized trials	serious ^b^	not serious	not serious	not serious	none	21/43 (48.8%)	22/43 (51.2%)	not estimable		⨁⨁⨁ ◯ Moderate ^b^	IMPORTANT
Effects of progressive elastic band resistance exercise for aged osteosarcopenic adipose women												
1	randomized trials	not serious	not serious	not serious	not serious	none	15/27 (55.6%)	12/27 (44.4%)	not estimable		⨁⨁⨁⨁ High	IMPORTANT
The Favorable Effects of a High-Intensity Resistance Training on Sarcopenia in Older Community-Dwelling Men with Osteosarcopenia: The Randomized Controlled FrOST Study												
1	randomized trials	serious ^b^	not serious	not serious	not serious	none	19/40 (47.5%)	21/40 (52.5%)	not estimable		⨁⨁⨁ ◯ Moderate ^b^	IMPORTANT

Abbreviations: CI: confidence interval; ^a^: high; ^b^: some concerns.

## Data Availability

The datasets generated during and/or analyzed during the current research are available from the corresponding author upon reasonable request.

## Supplementary Materials

The following supporting information can be downloaded at: https://www.mdpi.com/article/10.3390/nu17172852/s1.
