# Supplementary material for: Effects of Strength Training on Body Composition, Physical Performance, and Protein or Calcium Intake in Older People with Osteosarcopenia: A Meta-Analysis
Source: Nutrients. 2025 Sep 2;17(17):2852. doi: 10.3390/nu17172852 (PMC12430211; doi:10.3390/nu17172852)
Supplement: Supplementary file 1 [file nutrients-17-02852-s001.zip › nutrients-3842181-supplementary.pdf]

Supplementary Material

Body composition

Study name	Hedges's g	Standard error	Variance	Lower limit	Upper limit	Z-Value	p-Value
Banitalebi et al. [33]	0,365	0,251	0,063	-0,127	0,857	1,453	0,146
Banitalebi et al. [34]	0,063	0,249	0,062	-0,425	0,551	0,254	0,800
Lee et al. [38]	0,293	0,378	0,143	-0,447	1,033	0,775	0,438
Kemmler et al. [36]	0,526	0,359	0,129	-0,177	1,230	1,466	0,143
Cunha et al. [35]a	0,000	0,303	0,092	-0,593	0,593	0,000	1,000
Cunha et al. [35]b	0,219	0,311	0,097	-0,390	0,829	0,705	0,481
	0,224	0,121	0,015	-0,014	0,461	1,844	0,065

(Statistics for each study)

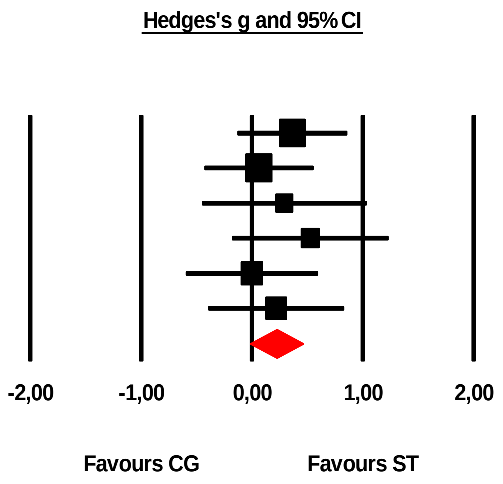

Figure S1. Forest plot of changes in BMD in older people with osteosarcopenia participating in strength training compared with older people with osteosarcopenia assigned as controls. Values shown are effect sizes (Hedges' g) with 95% confidence intervals (CI). The size of the squares plotted reflects the statistical weight of each study.

Study name	Hedges's g	Standard error	Variance	Lower limit	Upper limit	Z-Value	p-Value
Kemmler et al. [37]	-0,807	0,312	0,097	-1,418	-0,196	-2,587	0,010
Banitalebi et al. [33]	1,974	0,305	0,093	1,376	2,571	6,476	0,000
Banitalebi et al. [34]	1,178	0,270	0,073	0,648	1,707	4,360	0,000
Lee et al. [38]	0,234	0,377	0,142	-0,505	0,972	0,620	0,535
Cunha et al. [35]a	0,312	0,305	0,093	-0,285	0,910	1,026	0,305
Cunha et al. [35]b	0,933	0,316	0,100	0,314	1,552	2,953	0,003
	0,644	0,392	0,154	-0,125	1,412	1,641	0,101

(Statistics for each study)

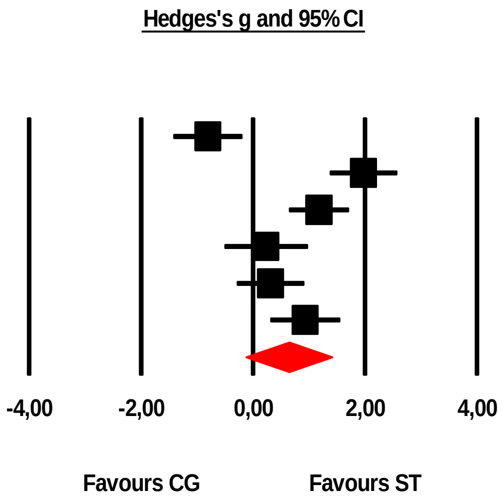

Figure S2. Forest plot of changes in BFP in older people with osteosarcopenia participating in strength training compared with older people with osteosarcopenia assigned as controls. Values shown are effect sizes (Hedges' g) with 95% confidence intervals (CI). The size of the squares plotted reflects the statistical weight of each study.

Study name	Hedges's g	Standard error	Variance	Lower limit	Upper limit	Z-Value	p-Value
	Statistics for each study						
Kemmler et al. [36]	2,788	0,424	0,180	1,956	3,620	6,570	0,000
Lee et al. [38]	0,244	0,377	0,142	-0,495	0,983	0,646	0,518
Lichtenberg et al. [39]	2,484	0,478	0,228	1,548	3,420	5,201	0,000
Cunha et al. [35]a	1,000	0,322	0,104	0,369	1,630	3,106	0,002
Cunha et al. [35]b	1,073	0,321	0,103	0,444	1,703	3,343	0,001
	1,484	0,443	0,196	0,616	2,352	3,352	0,001

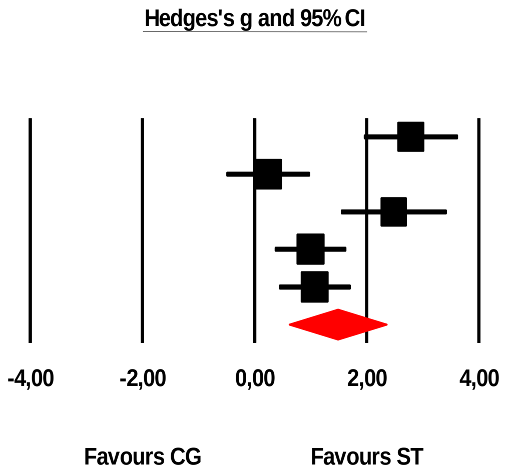

Figure S3. Forest plot of changes in SMI in older people with osteosarcopenia participating in strength training compared with older people with osteosarcopenia assigned as controls. Values shown are effect sizes (Hedges' g) with 95% confidence intervals (CI). The size of the squares plotted reflects the statistical weight of each study.

Physical performance

Study name	Hedges's g	Standard error	Variance	Lower limit	Upper limit	Z-Value	p-Value
	Statistics for each study						
Banitalebi et al. [33]	2,621	0,341	0,116	1,952	3,289	7,679	0,000
Lee et al. [38]	0,491	0,381	0,146	-0,257	1,239	1,287	0,198
Lichtenberg et al. [39]	0,949	0,328	0,108	0,307	1,592	2,895	0,004
	1,359	0,644	0,415	0,097	2,621	2,111	0,035

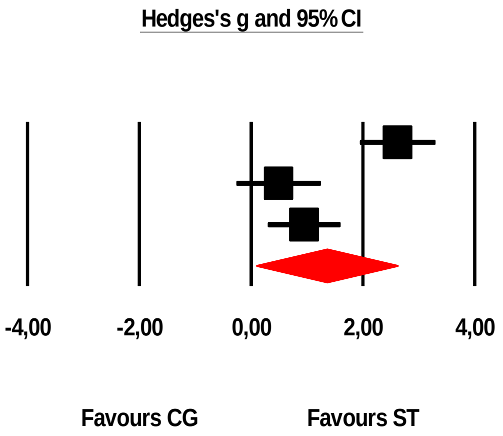

Figure S4. Forest plot of changes in MIHS in older people with osteosarcopenia participating in strength training compared with older people with osteosarcopenia assigned as controls. Values shown are effect sizes (Hedges' g) with 95% confidence intervals (CI). The size of the squares plotted reflects the statistical weight of each study.

Study name	Hedges's g	Standard error	Variance	Lower limit	Upper limit	Z-Value	p-Value
	Statistics for each study						
Banitalebi et al. [33]	0,424	0,252	0,063	-0,070	0,917	1,683	0,092
Lee et al. [38]	0,209	0,377	0,142	-0,530	0,947	0,554	0,580
Lichtenberg et al. [39]	0,251	0,312	0,097	-0,359	0,862	0,807	0,420
	0,324	0,174	0,030	-0,016	0,665	1,867	0,062

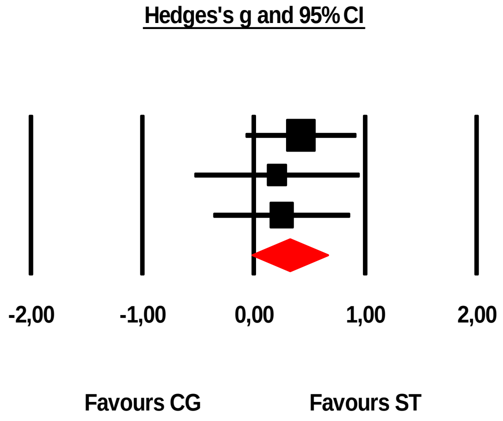

Figure S5. Forest plot of changes in gait speed in older people with osteosarcopenia participating in strength training compared with older people with osteosarcopenia assigned as controls. Values shown are effect sizes (Hedges' g) with 95% confidence intervals (CI). The size of the squares plotted reflects the statistical weight of each study.

Intake macro nutrients

Study name	Statistics for each study						
	Hedges's g	Standard error	Variance	Lower limit	Upper limit	Z-Value	p-Value
Kemmler et al. [36]	0,805	0,312	0,097	0,194	1,416	2,581	0,010
Banitalebi et al. [33]	2,171	0,315	0,099	1,553	2,789	6,887	0,000
Kemmler et al. [37]	0,067	0,300	0,090	-0,520	0,654	0,224	0,823
Lichtenberg et al. [39]	0,648	0,308	0,095	0,045	1,250	2,106	0,035
	0,919	0,443	0,196	0,051	1,788	2,076	0,038

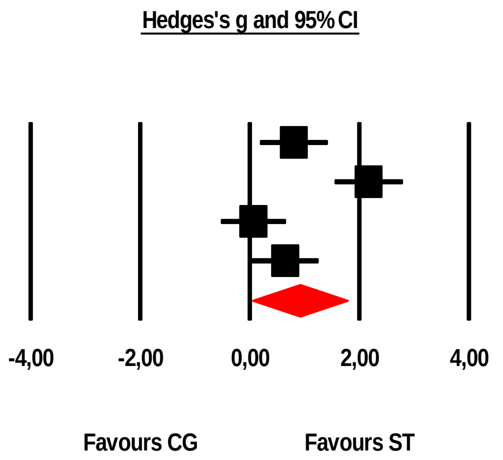

Figure S6. Forest plot of changes in protein intake in older people with osteosarcopenia participating in strength training compared with older people with osteosarcopenia assigned as controls. Values shown are effect sizes (Hedges' g) with 95% confidence intervals (CI). The size of the squares plotted reflects the statistical weight of each study.

Study name	Statistics for each study						
	Hedges's g	Standard error	Variance	Lower limit	Upper limit	Z-Value	p-Value
Kemmler et al. [36]	0,354	0,302	0,091	-0,238	0,945	1,172	0,241
Banitalebi et al. [33]	0,189	0,249	0,062	-0,300	0,678	0,759	0,448
Kemmler et al. [37]	0,035	0,299	0,090	-0,552	0,622	0,116	0,908
	0,191	0,162	0,026	-0,126	0,509	1,183	0,237

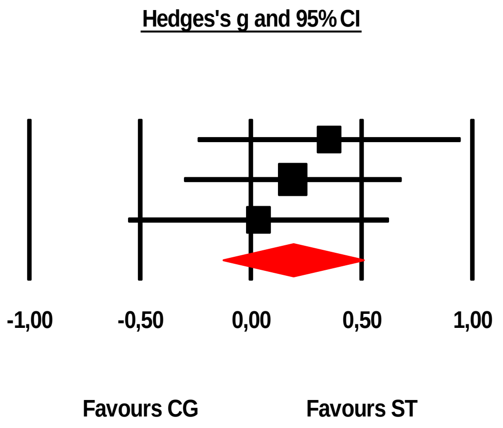

Figure S7. Forest plot of changes in calcium intake in older people with osteosarcopenia participating in strength training compared with older people with osteosarcopenia assigned as controls. Values shown are effect sizes (Hedges' g) with 95% confidence intervals (CI). The size of the squares plotted reflects the statistical weight of each study.

Study name	Subgroup within study	Hedges's g	Standard error	Variance	Lower limit	Upper limit	Z-Value	p-Value
Banitalebi et al. [33]	EBT	0,365	0,251	0,063	-0,127	0,857	1,453	0,146
Banitalebi et al. [34]	EBT	0,063	0,249	0,062	-0,425	0,551	0,254	0,800
Lee et al. [38]	EBT	0,293	0,378	0,143	-0,447	1,033	0,775	0,438
		0,227	0,160	0,026	-0,087	0,541	1,418	0,156
Kemmler et al. [37]	RT	0,856	0,368	0,136	0,134	1,579	2,324	0,020
Cunha et al. [35]a	RT	0,000	0,303	0,092	-0,593	0,593	0,000	1,000
Cunha et al. [35]b	RT	0,219	0,311	0,097	-0,390	0,829	0,705	0,481
		0,300	0,187	0,035	-0,067	0,666	1,603	0,109

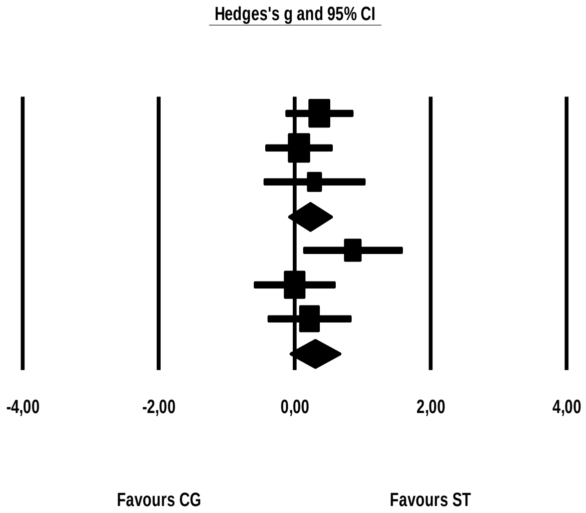

Figure S8. Forest plot of changes in sub group BMD in older people with osteosarcopenia participating in strength training compared with older people with osteosarcopenia assigned as controls. Values shown are effect sizes (Hedges' g) with 95% confidence intervals (CI). The size of the squares plotted reflects the statistical weight of each study.

Study name	Subgroup within study	Hedges's g	Standard error	Variance	Lower limit	Upper limit	Z-Value	p-Value
Banitalebi et al. [33]	EBT	1,974	0,305	0,093	1,376	2,571	6,476	0,000
Banitalebi et al. [34]	EBT	1,178	0,270	0,073	0,648	1,707	4,360	0,000
Lee et al. [38]	EBT	0,234	0,377	0,142	-0,505	0,972	0,620	0,535
		1,151	0,463	0,214	0,244	2,057	2,487	0,013
Cunha et al. [35]a	RT	0,312	0,305	0,093	-0,285	0,910	1,026	0,305
Cunha et al. [35]b	RT	0,933	0,316	0,100	0,314	1,552	2,953	0,003
Kemmler et al. [37]	RT	-0,807	0,312	0,097	-1,418	-0,196	-2,587	0,010
		0,146	0,505	0,255	-0,844	1,135	0,288	0,773

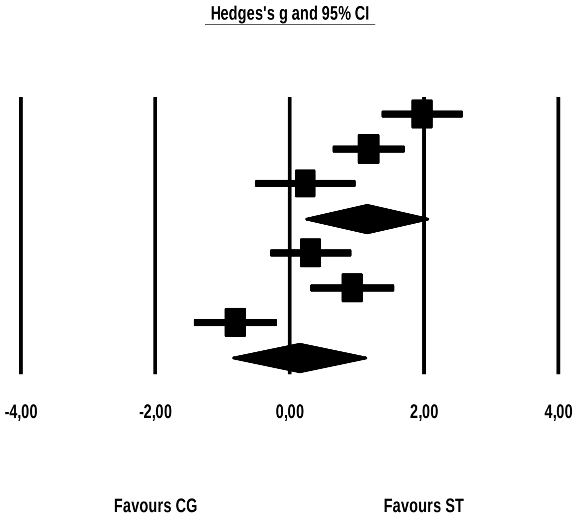

Figure S9. Forest plot of changes in sub group BFP in older people with osteosarcopenia participating in strength training compared with older people with osteosarcopenia assigned as controls. Values shown are effect sizes (Hedges' g) with 95% confidence intervals (CI). The size of the squares plotted reflects the statistical weight of each study.
